# The Individual Cup at Communion

**Published:** 1895-03

**Authors:** John L. Moffat

**Affiliations:** Secretary


### THE INDIVIDUAL CUP AT COMMUNION.

At the Forty-fourth Annual Meeting of the Homœopathic Medical Society of the State of New York, held in Albany February 12th and 13th, it was

*Resolved*, This Society is in full accord with the statement that the prevention of disease is preferable to its cure, and, believing that the adoption of sanitary methods tends to the prolongation of life and the prevention of disease, desires emphatically to place itself on record as being in perfect sympathy with the movement, now becoming widely spread, to dispense with the usual method of distributing the Communion wine, and urges the speedy adoption of the Individual Cup system, thereby avoiding the possibility of contracting many forms of infectious disease.

JOHN L. MOFFAT, M. D.,
*Secretary.*

